# Functional hypothalamic amenorrhea and anti-Müllerian hormone: insights for fertility assessment

**DOI:** 10.1007/s40618-026-02878-4

**Published:** 2026-05-06

**Authors:** Giovanna Notaristefano, Anna Tropea, Anastasiya Samasiuk, Monia Ranalli, Martina Asia Policriti, Alice Diterlizzi, Tullio Ghi, Antonio Lanzone, Rosanna Apa

**Affiliations:** 1 Department of Women’s and Children’s Health Sciences and Public Health, Fondazione Policlinico Universitario A. Gemelli, IRCCS, L.go Agostino Gemelli 8, 00168 Rome, Italy; 2 Università Cattolica del Sacro Cuore, Largo F. Vito 1, 00168 Rome, Italy; 3 Department of Statistical Sciences, Sapienza University of Rome, 00185 Rome, Italy

**Keywords:** Anti-Müllerian hormone, Functional hypothalamic amenorrhea, Ovarian reserve, Hormone replacement therapy, Polycystic ovary syndrome

## Abstract

**Background:**

Anti-Müllerian hormone (AMH) is widely used as a marker of ovarian reserve. However, its reliability in functional hypothalamic amenorrhea (FHA) remains debated. FHA is characterized by reduced gonadotropin secretion and impaired folliculogenesis, which may influence AMH expression and lead to an underestimation of reproductive potential.

**Methods:**

We conducted a case–control study including women diagnosed with FHA and age-matched healthy controls. FHA patients were stratified according to the presence or absence of polycystic ovarian morphology (PCOM). Serum gonadotropins, estradiol, androgens, and AMH were measured and compared between groups. Correlation analyses were performed to explore associations among hormonal parameters.

**Results:**

Overall, AMH concentrations in FHA patients were comparable to those of controls. However, stratification revealed that FHA without PCOM was associated with significantly reduced AMH, whereas FHA with PCOM displayed increased values. FHA patients also showed reduced gonadotropins, estradiol, and androgens compared with controls. In the non-PCOM FHA subgroup, AMH correlated positively with androgens and negatively with estradiol. These findings suggest that suppression of gonadotropins and consequent androgen deficiency may contribute to reduced AMH levels in FHA.

**Conclusions:**

This pilot study indicates that low AMH in FHA may reflect hypothalamic suppression of gonadotropins rather than true ovarian reserve potentially leading to misinterpretation in fertility counseling. These observations should be interpreted with caution given the small sample size, but they support the hypothesis that AMH levels might recover with restoration of hypothalamic–pituitary activity and menstrual cycles. Larger, longitudinal studies are warranted to confirm the reversibility of AMH and clarify its prognostic value in FHA.

**Graphical abstract:**

Proposed mechanism linking hypothalamic suppression to reduced AMH levels in functional hypothalamic amenorrhea (FHA). Reduced GnRH pulsatility leads to decreased LH secretion and consequent reduction in ovarian androgen production. The resulting hypoandrogenic environment impairs granulosa cell function and attenuates AMH production, reflecting a functional and potentially reversible suppression rather than an irreversible depletion of ovarian reserve.

**Figure:**
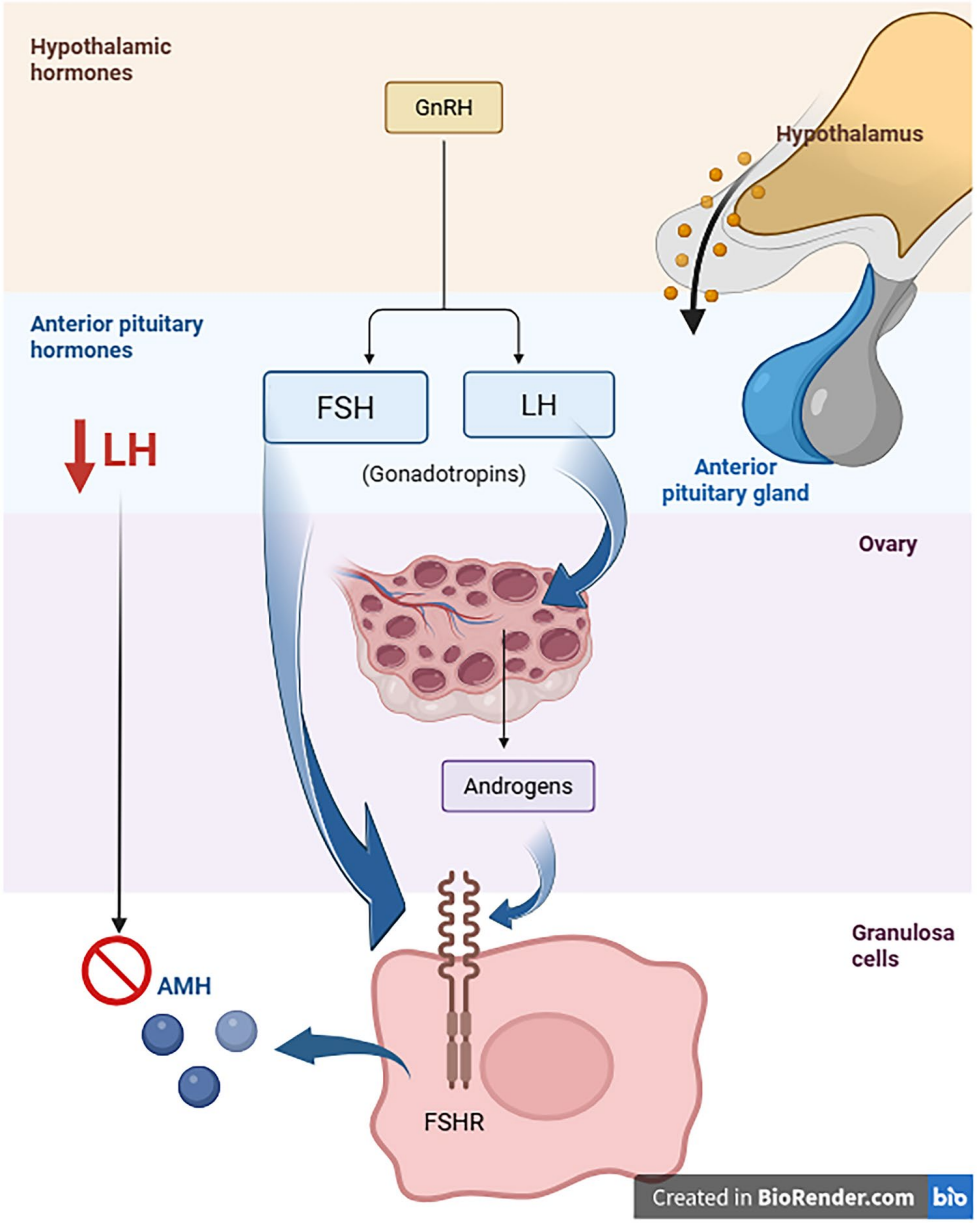

## Introduction

Anti-Müllerian hormone (AMH) is a glycoprotein belonging to the transforming growth factor-β (TGF-β) superfamily. In females, it is secreted by granulosa cells of growing follicles from the primary to the small antral stage, and its expression declines after follicle selection induced by follicle-stimulating hormone (FSH) [1, 2]. Because it reflects the number of growing follicles, AMH has been widely adopted as a surrogate marker of ovarian reserve, with strong correlations to the antral follicle count (AFC) [3, 4]. AMH may also modulate GnRH neuron activity, suggesting a bidirectional interaction with gonadotropin secretion [5]. These findings highlight a potential bidirectional relationship between AMH and gonadotropin activity.

Recent studies have demonstrated that AMH is not completely stable and may vary across physiological and pharmacological contexts, including menstrual cycle fluctuations, pregnancy, hormonal contraception, and states of gonadotropin suppression [6–9]. AMH levels are also influenced by constitutional factors such as BMI, with lower adiposity being associated with higher AMH concentrations in epidemiological studies [10, 11]. This inverse relationship is particularly relevant in FHA, where low BMI contributes to altered gonadotropin release and chronic anovulation, reinforcing the bidirectional interaction between AMH and hypothalamic–pituitary function Severe gonadotropin suppression has been associated with reduced AMH levels [12, 13]. These data suggest that AMH does not merely reflect the follicle pool but also its responsiveness to gonadotropin stimulation.

Functional hypothalamic amenorrhea (FHA) is the most common acquired form of hypogonadotropic hypogonadism, accounting for 15–30% of cases [14]. It is caused by reduced GnRH pulsatility, typically triggered by energy imbalance, excessive physical activity, or psychological stress [15]. FHA is characterized by anovulation, hypoestrogenism, and low gonadotropin levels [16]. Despite this endocrine milieu, studies on AMH in FHA have yielded conflicting results: some report normal or elevated levels [17–19], while others describe reduced concentrations [20].

A major confounding factor is the frequent presence of polycystic ovarian morphology (PCOM), reported in up to 50% of FHA cases [21, 22]. Since PCOM is associated with higher AMH, its inclusion may mask the reduction expected in FHA patients without PCOM.

Taken together, these uncertainties raise important questions about the validity of AMH as a marker of ovarian reserve in FHA. The reliability of AMH as an ovarian reserve marker may be limited by the fact that it reflects the cohort of small antral follicles responsive to gonadotropins, rather than the overall primordial follicle pool. In clinical contexts characterized by sustained gonadotropin suppression, AMH levels may therefore be reduced, potentially underestimating the actual ovarian reserve. A better understanding of AMH dynamics in this condition is crucial for appropriate fertility counseling and management.

The present study aimed to investigate AMH levels in women with FHA compared with age-matched controls, disentangling the influence of PCOM, and to evaluate the clinical utility of AMH as an ovarian reserve marker in this population.

## Materials and methods

### Study design

This was a monocentric, prospective, longitudinal observational study conducted at the Department of Women’s and Children’s Health Sciences and Public Health, Fondazione Policlinico Universitario A. Gemelli IRCCS, Rome. The study protocol was approved by the institutional ethics committee (Prot. N. 0019027/23, June 16, 2023; ID 5321), and all participants provided written informed consent. The study was performed in accordance with the Declaration of Helsinki (2013 revision).

### Population

Seventy-four women diagnosed with functional hypothalamic amenorrhea (FHA) and 51 healthy controls were initially recruited. FHA was defined as secondary amenorrhea lasting ≥6 months, with estradiol <50 pg/ml, FSH and LH <10 mIU/ml, and a negative medroxyprogesterone acetate (MAP) withdrawal test, after exclusion of organic, structural, or systemic causes. Exclusion criteria were: hormonal therapy or drug abuse within the preceding 3 months, autoimmune disease, diabetes mellitus, polycystic ovary syndrome (PCOS), major surgery within 3 months, hepatic, intestinal, or other endocrine dysfunctions, and a positive MAP test.

Controls were healthy women with regular menstrual cycles, matched for age and subjected to the same exclusion criteria. The presence of polycystic ovarian morphology (PCOM) was also an exclusion criterion for controls. PCOM was defined according to the 2023 international guideline as ≥20 follicles in at least one ovary and/or ovarian volume ≥10 ml, in the absence of a dominant follicle (Teede et al., 2023). Although antral follicle count (AFC) was not formally recorded in the dataset because pelvic ultrasound examinations were performed during routine clinical practice, follicle number was systematically evaluated by the operators and directly used to diagnose polycystic ovarian morphology (PCOM) in accordance with the 2023 International Evidence-Based Guideline. Polycystic ovarian morphology (PCOM) was assessed using both recommended criteria—follicle number and ovarian volume—as all three ovarian diameters were routinely measured for each ovary. Transvaginal ultrasound examinations were performed using high-resolution equipment (Voluson E8/E10 systems, GE Healthcare) equipped with 5–9 MHz endovaginal probes. All scans were conducted by three experienced operators trained within the same institution and following identical scanning protocols, ensuring a uniform methodological approach.

Because AMH levels are strongly age-dependent, a rigorous age matching was performed between FHA patients and controls. This resulted in a final study cohort of 46 FHA patients and 46 age-matched controls. Within the FHA group, 13 women (28%) showed PCOM and were analyzed separately, leaving 33 women with FHA only.

### Clinical and laboratory assessment

At enrollment, all participants underwent standardized clinical evaluation, including pelvic ultrasonography, anthropometric measurements, nutritional assessment, and psychological consultation. Body mass index (BMI) was calculated as weight (kg)/height (m^2^). After an overnight fast, venous blood samples were collected for hormone assays, including AMH, testosterone, dehydroepiandrosterone-sulfate (DHEAS), androstenedione, 17-hydroxyprogesterone (17OHP), sex hormone-binding globulin (SHBG), FSH, LH, and estradiol. Assays were performed in the institutional laboratory with commercially available automated immunoassays; intra- and inter-assay coefficients of variation were <10%. Serum AMH concentrations were measured using a fully automated chemiluminescent immunoassay (Access AMH, Beckman Coulter, Brea, CA, USA). The analytical sensitivity of the assay was 0.01 ng/mL, with intra- and inter-assay coefficients of variation below 5%. All samples were processed according to the manufacturer’s instructions and analyzed in duplicate after a single thaw.

Laboratory reference ranges were those provided by the institutional laboratory of the Fondazione Policlinico Universitario A. Gemelli IRCCS and are reported according to the units used in the tables. In women of reproductive age, reference values were as follows: FSH 3.5–10.0 IU/L (follicular phase); LH 2.0–10.0 IU/L (follicular phase); estradiol 20–80 pg/mL (early follicular phase); total testosterone 0.15–0.45 nmol/L; DHEAS 100–350 µg/dL; androstenedione 1.0–3.0 nmol/L; 17OHP 0.2–1.0 ng/mL (follicular phase); SHBG 20–120 nmol/L; AMH 0.5–8.0 ng/mL.

Data were collected and managed using REDCap electronic data capture tools hosted at Fondazione Policlinico Universitario A. Gemelli IRCCS [23, 24].

### Statistical analysis

Statistical analyses were performed with R (version 4.0.5). Continuous variables were expressed as mean ± standard deviation and median with interquartile range (IQR). Comparisons between FHA patients and controls were performed using non-parametric tests (Wilcoxon rank-sum). Subgroup analyses compared FHA without PCOM, FHA with PCOM, and controls. Univariate associations between variables were tested using Spearman’s correlation. No adjustment for multiple testing was applied, given the exploratory nature of the study. Statistical significance was set at p<0.05.

## Results

A total of 46 women with FHA and 46 age-matched controls were included in the final analysis. Among FHA patients, 13 (28%) showed polycystic ovarian morphology (PCOM) on ultrasound and were categorized as FHA+PCOM, while the remaining 33 (72%) were classified as FHA without PCOM.

When all FHA patients were compared with controls, no significant differences in AMH concentrations were observed (3.29 ± 1.82 vs 3.81 ± 2.00 ng/ml, p=0.28). However, as expected, FHA patients displayed significantly lower BMI, gonadotropins (LH, FSH), estradiol, testosterone, and 17-hydroxyprogesterone (all p<0.01), while SHBG and DHEAS did not differ significantly (Table 1).

**Table 1:** Clinical characteristics and hormonal values of all patients with FHA versus healthy controls

	FHA all (n=46)		Controls (n=46)		*p*-value
	Mean ± SD	Median (IQR)	Mean ± SD	Median (IQR)	
Age (years)	25.39 ± 3.64	24.00 (3.50)	25.39 ± 3.64	24.00 (3.50)	1.00
BMI (kg/m^2^)	19.74 ± 2.20	19.50 (3.30)	21.47 ± 2.65	21.25 (2.20)	0.01*
AMH (ng/ml)	3.29 ± 1.82	3.56 (3.40)	3.81 ± 2.00	3.00 (2.77)	0.28
T (nmol/L)	0.24 ± 0.09	0.23 (0.11)	0.29 ± 0.08	0.28 (0.10)	0.01*
DHEAS (µg/dL)	2071 ± 1168	1807 (1124)	2366 ± 1210	2139 (1054)	0.27
A (nmol/L)	1.29 ± 0.73	1.30 (0.46)	1.54 ± 0.51	1.40 (0.58)	0.08
17OHP (ng/ml)	0.30 ± 0.16	0.30 (0.15)	0.43 ± 0.17	0.40 (0.23)	< 0.01*
SHBG (nmol/L)	66.63 ± 33.45	58.00 (38.5)	75.97 ± 29.25	71.5 (36.25)	0.07
LH (UI/ml)	1.27 ± 1.24	0.90 (1.20)	5.05 ± 1.91	5.00 (1.80)	< 0.0001*
FSH (UI/ml)	5.51 ± 2.07	5.80 (2.65)	6.82 ± 1.47	6.65 (2.35)	0.01*
E2 (pg/ml)	32.71 ± 19.34	26 (19)	58.89 ± 22.99	56 (15.25)	< 0.0001*

FHA = Functional Hypothalamic Amenorrhea; SD = Standard Deviation; BMI = Body Mass Index; AMH = Anti-Müllerian Hormone; T = Testosterone; DHEAS = Dehydroepiandrosterone-Sulfate; A = Androstenedione; 17OHP = 17-hydroxyprogesterone; SHBG = Sex Hormone-Binding Globulin; LH = Luteinizing Hormone; FSH = Follicle-Stimulating Hormone; E2 = Estradiol

^*^Bold values identify statistical significance (5%, *p* < 0.05)

Stratified analyses revealed a contrasting picture. In FHA without PCOM, AMH levels were significantly reduced compared with controls (2.64 ± 1.62 vs 3.81 ± 2.00 ng/ml, p=0.01). This group also showed lower BMI, androgens, gonadotropins, and estradiol (all p<0.05) (Table 2). This profile reflects not only the hypoestrogenic–hypogonadotropic state observed in all FHA patients, but also the more pronounced androgen deficiency that uniquely characterizes the FHA without PCOM subgroup. By contrast, FHA+PCOM patients exhibited markedly higher AMH than controls (5.11 ± 1.05 vs 3.81 ± 2.00 ng/ml, p=0.02), despite persistently low LH and estradiol (p<0.01) (Table 3). Direct comparison between FHA subgroups confirmed that AMH was significantly lower in FHA without PCOM than in FHA+PCOM (2.64 ± 1.62 vs 5.11 ± 1.05 ng/ml, p<0.001), accompanied by a lower BMI and reduced androstenedione (Table 4). The duration of amenorrhea did not differ significantly between patients with FHA without PCOM and those with FHA+PCOM (Table 4).

**Table 2:** Clinical characteristics and hormonal values of patients with FHA without PCOM versus healthy controls

	FHA without PCOM (n=33)		Controls (n=46)		*p*-value
	Mean ± SD	Median (IQR)	Mean ± SD	Median (IQR)	
Age (years)	25.69 ± 3.88	24.5 (5.75)	25.39 ± 3.64	24 (3.5)	0.79
BMI (kg/m^2^)	19.15 ± 1.85	18.95 (2.92)	21.47 ± 2.65	21.25 (2.2)	0.001*
AMH (ng/ml)	2.64 ± 1.62	1.88 (3.16)	3.81 ± 2.00	3 (2.77)	0.01*
T (nmol/L)	0.23 ± 0.08	0.22 (0.09)	0.29 ± 0.08	0.28 (0.1)	< 0.01*
DHEAS (µg/dL)	1949 ± 1162	1807 (1227)	2366 ± 1210	2139 (1054)	0.16
A (nmol/L)	1.14 ± 0.67	1.2 (0.5)	1.54 ± 0.51	1.4 (0.58)	0.02*
17OHP (ng/ml)	0.28 ± 0.14	0.3 (0.1)	0.43 ± 0.17	0.4 (0.23)	< 0.01*
SHBG (nmol/L)	67.64 ± 38.12	58 (43)	75.97 ± 29.25	71.5 (36.25)	0.13
LH (UI/ml)	1.12 ± 1.27	0.7 (1.3)	5.05 ± 1.91	5 (1.8)	< 0.0001*
FSH (UI/ml)	5.48 ± 2.33	5.9 (3)	6.82 ± 1.47	6.65 (2.35)	0.04*
E2 (pg/ml)	31.33 ± 20.54	24.5 (15)	58.89 ± 22.99	56 (15.25)	< 0.0001*

FHA = Functional Hypothalamic Amenorrhea; SD = Standard Deviation; BMI = Body Mass Index; AMH = Anti-Müllerian Hormone; T = Testosterone; DHEAS = Dehydroepiandrosterone-Sulfate; A = Androstenedione; 17OHP = 17-hydroxyprogesterone; SHBG = Sex Hormone-Binding Globulin; LH = Luteinizing Hormone; FSH = Follicle-Stimulating Hormone; E2 = Estradiol

^*^Bold values identify statistical significance (5%, *p* < 0.05)

**Table 3:** Clinical characteristics and hormonal values of patients with overlap FHA + PCOM versushealthy controls

	FHA+PCOM (n=13)		Controls (n=46)		*p*-value
	Mean ± SD	Median (IQR)	Mean ± SD	Median (IQR)	
Age (years)	24.44 ± 3.13	23 (2)	25.39 ± 3.64	24 (3.5)	0.46
BMI (kg/m^2^)	21.53 ± 2.2	21.2 (3.1)	21.47 ± 2.65	21.25 (2.2)	0.87
AMH (ng/ml)	5.11 ± 1.05	5.29 (2.03)	3.81 ± 2.00	3 (2.77)	0.02*
T (nmol/L)	0.28 ± 0.08	0.25 (0.1)	0.29 ± 0.08	0.28 (0.1)	0.85
DHEAS (µg/dL)	2570 ± 1078	2051 (1635)	2366 ± 1210	2139 (1054)	0.62
A (nmol/L)	1.79 ± 0.72	1.5 (0.9)	1.54 ± 0.51	1.4 (0.58)	0.47
17OHP (ng/ml)	0.43 ± 0.21	0.4 (0.4)	0.43 ± 0.17	0.4 (0.23)	0.89
SHBG (nmol/L)	61.67 ± 17.78	58 (19)	75.97 ± 29.25	71.5 (36.25)	0.10
LH (UI/ml)	1.73 ± 1.16	1.3 (1)	5.05 ± 1.91	5 (1.8)	< 0.0001*
FSH (UI/ml)	5.7 ± 1.38	5.8 (0.7)	6.82 ± 1.47	6.65 (2.35)	0.055
E2 (pg/ml)	37.67 ± 16.72	35 (21)	58.89 ± 22.99	56 (15.25)	< 0.01*

FHA = Functional Hypothalamic Amenorrhea; SD = Standard Deviation; BMI = Body Mass Index; AMH = Anti-Müllerian Hormone; T = Testosterone; DHEAS = Dehydroepiandrosterone-Sulfate; A = Androstenedione; 17OHP = 17-hydroxyprogesterone; SHBG = Sex Hormone-Binding Globulin; LH = Luteinizing Hormone; FSH = Follicle-Stimulating Hormone; E2 = Estradiol

^*^Bold values identify statistical significance (5%, *p* < 0.05)

**Table 4:** Clinical characteristics and hormonal values of patients with FHA without PCOM compared with patients with FHA + PCOM

	FHA without PCOM (n=33)		FHA+PCOM (n=13)		*p*-value
	Mean ± SD	Median (IQR)	Mean ± SD	Median (IQR)	
Age (years)	25.69 ± 3.88	24.5 (5.75)	24.44 ± 3.13	23 (2)	0.39
BMI (kg/m^2^)	19.15 ± 1.85	18.95 (2.92)	21.53 ± 2.2	21.2 (3.1)	0.01*
AMH (ng/ml)	2.64 ± 1.62	1.88 (3.16)	5.11 ± 1.05	5.29 (2.03)	< 0.001*
T (nmol/L)	0.23 ± 0.08	0.22 (0.09)	0.28 ± 0.08	0.25 (0.1)	0.09
DHEAS (µg/dL)	1949 ± 1162	1807 (1227)	2570 ± 1078	2051 (1635)	0.15
A (nmol/L)	1.14 ± 0.67	1.2 (0.5)	1.79 ± 0.72	1.5 (0.9)	0.03*
17OHP (ng/ml)	0.28 ± 0.14	0.3 (0.1)	0.43 ± 0.21	0.4 (0.4)	0.12
SHBG (nmol/L)	67.64 ± 38.12	58 (43)	61.67 ± 17.78	58 (19)	0.71
LH (UI/ml)	1.12 ± 1.27	0.7 (1.3)	1.73 ± 1.16	1.3 (1)	0.07
FSH (UI/ml)	5.48 ± 2.33	5.9 (3)	5.7 ± 1.38	5.8 (0.7)	0.68
E2 (pg/ml)	31.33 ± 20.54	24.5 (15)	37.67 ± 16.72	35 (21)	0.14
Months of amenorrhea (months)	17.47 ± 15.03	12.00 (15)	21.00 ± 24.68	14.00 (14.5)	0.53

FHA = Functional Hypothalamic Amenorrhea; SD = Standard Deviation; BMI = Body Mass Index; AMH = Anti-Müllerian Hormone; T = Testosterone; DHEAS = Dehydroepiandrosterone-Sulfate; A = Androstenedione; 17OHP = 17-hydroxyprogesterone; SHBG = Sex Hormone-Binding Globulin; LH = Luteinizing Hormone; FSH = Follicle-Stimulating Hormone; E2 = Estradiol

^*^Bold values identify statistical significance (5%, *p* < 0.05)

**Fig. 2:**
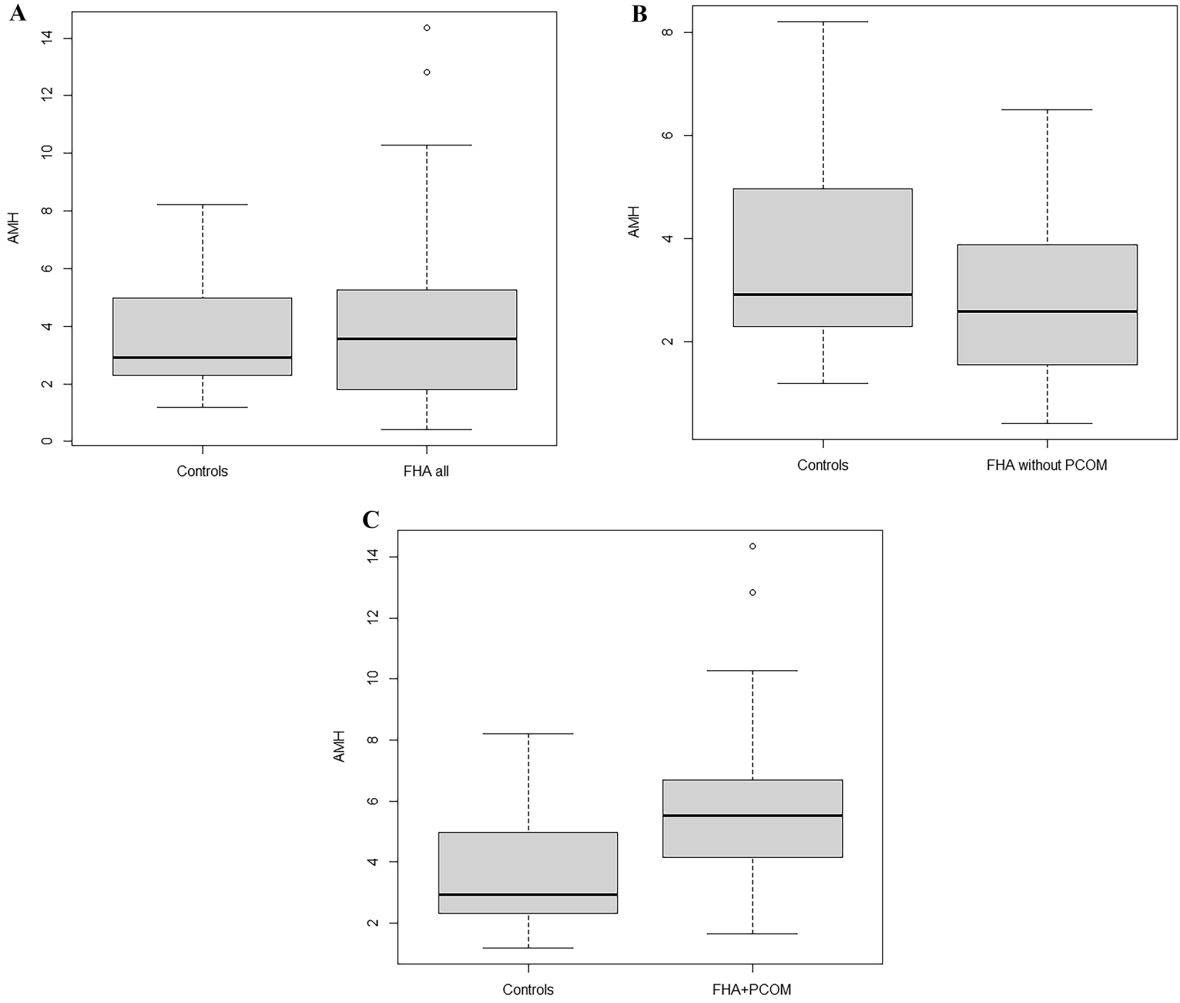
Serum AMH concentrations across study groups. (**A**) Comparison of serum AMH levels between healthy controls and all patients with functional hypothalamic amenorrhea (FHA). (**B**) Comparison of serum AMH levels between healthy controls and patients with FHA without polycystic ovarian morphology (PCOM). (**C**) Comparison of serum AMH levels between healthy controls and patients with FHA+PCOM. Boxes represent medians and interquartile ranges; whiskers indicate the 5th–95th percentiles. Abbreviations: AMH, anti-Müllerian hormone; FHA, functional hypothalamic amenorrhea; PCOM, polycystic ovarian morphology

**Fig. 1:**
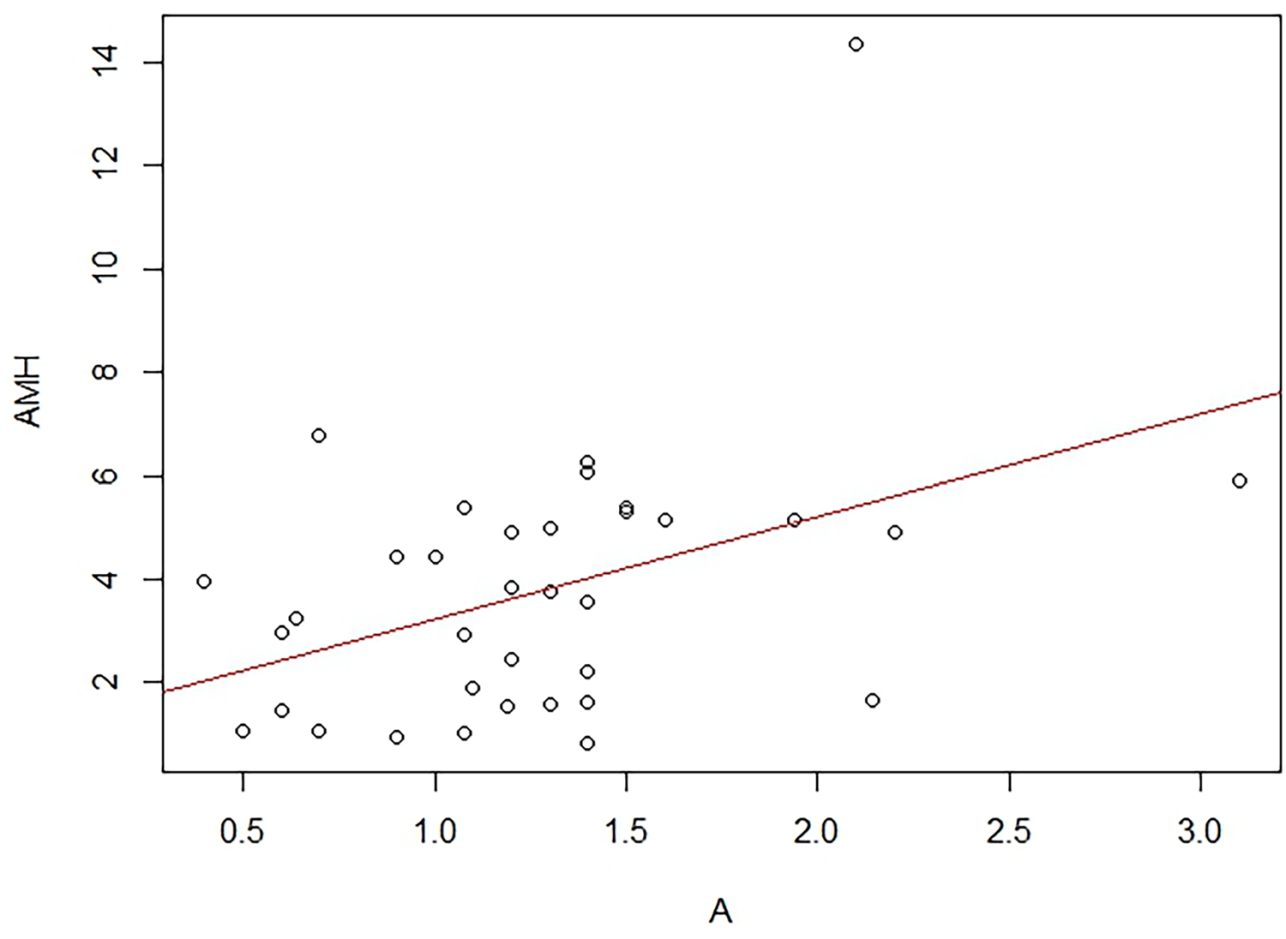
Correlations between AMH and circulating androgens in FHA. Scatter plots illustrate the relationships between serum AMH concentrations and androgen levels (androstenedione, testosterone, and DHEAS) in the overall FHA cohort. Correlations were assessed using Spearman’s rank correlation coefficient. Abbreviations: AMH, anti-Müllerian hormone; DHEAS, dehydroepiandrosterone sulfate

Correlation analyses provided further insight into the hormonal interplay. In the whole FHA cohort, AMH positively correlated with androgens (androstenedione, testosterone, DHEAS) and negatively with SHBG, while LH correlated with estradiol and androgens (Table 5). These associations between AMH and androgens are further illustrated in Fig. 1. When restricted to FHA without PCOM, AMH maintained a positive correlation with androgens but also showed a negative correlation with estradiol (Table 6). In FHA+PCOM, correlations shifted: AMH no longer associated with other hormones, while strong intercorrelations emerged among androgens, LH, and BMI (Table 7).

**Table 5:** Spearman correlation matrix in the FHA whole group

	AMH	FSH	LH	BMI	E2	A	T	DHEAS	17OHP	SHBG
AMH (ng/ml)	1									
FSH (UI/ml)	-0.27	1								
LH (UI/ml)	0.04	0.52*	1							
BMI (kg/m^2^)	0.09	-0.05	0.33*	1						
E2 (pg/ml)	-0.08	-0.03	0.3*	0.16	1					
A (nmol/L)	0.43*	0.25	0.43*	0.22	0.18	1				
T (nmol/L)	0.33*	-0.07	0.21	0.17	0.21	0.65*	1			
DHEAS (µg/dL)	0.35*	-0.03	0.18	0.15	0.08	0.67*	0.69*	1		
17OHP (ng/ml)	0.18	0.28	0.6*	0.24	0.3	0.64*	0.63*	0.59*	1	
SHBG (nmol/L)	-0.09	-0.12	-0.37*	-0.18	0.4	-0.29*	0.88	-0.17	-0.35*	1
Months of amenorrhea	0.19	-0.06	0.04	-0.01	-0.3*	0.08	0.06	-0.01	0.23	-0.33*

A negative correlation is denoted by a minus sign, while positive correlations are indicated otherwise

^*^Statistically significant correlations are highlighted in bold (p-value < 0.05)

AMH, anti-Mullerian hormone; FSH, follicle-stimulating hormone; LH, luteinizing hormone; BMI, body mass index; E2, estradiol; A, androstenedione; T,testosterone; DHEAS, dehydroepiandrosterone-sulfate; 17OHP, 17-hydroxyprogesterone; SHBG, sex hormone-binding globulin

**Table 6:** Spearman correlation matrix in the FHA group without PCOM patients

	AMH	FSH	LH	BMI	E2	A	T	DHEAS	17OHP	SHBG
AMH (ng/ml)	1									
FSH (UI/ml)	-0.17	1								
LH (UI/ml)	0.06	0.54*	1							
BMI (kg/m^2^)	-0.15	0.04	0.23	1						
E2 (pg/ml)	-0.41*	0.07	0.22	-0.07	1					
A (nmol/L)	0.39*	0.2	0.33	0.12	-0.03	1				
T (nmol/L)	0.48*	-0.18	0.01	-0.1	-0.05	0.67*	1			
DHEAS (µg/dL)	0.41*	-0.14	-0.04	-0.14	-0.09	0.63*	0.7*	1		
17OHP (ng/ml)	0.26	0.36	0.58*	-0.14	0.07	0.75*	0.57*	0.52*	1	
SHBG (nmol/L)	-0.13	-0.22	-0.37*	0.03	0.12	-0.31	-0.01	0.05	-0.4	1
Months of amenorrhea	0.15	0.03	0.04	-0.14	-0.32	0.24	0.15	-0.05	0.43*	-0.28

A negative correlation is denoted by a minus sign, while positive correlations are indicated otherwise

^*^Statistically significant correlations are highlighted in bold (p-value < 0.05)

AMH, anti-Mullerian hormone; FSH, follicle-stimulating hormone; LH, luteinizing hormone; BMI, body mass index; E2, estradiol; A, androstenedione; T,testosterone; DHEAS, dehydroepiandrosterone-sulfate; 17OHP, 17-hydroxyprogesterone; SHBG, sex hormone-binding globulin

**Table 7:** Spearman correlation matrix in the patient group with overlap of FHA and PCOS

	AMH	FSH	LH	BMI	E2	A	T	DHEAS	17OHP	SHBG
AMH (ng/ml)	1									
FSH (UI/ml)	-0.19	1								
LH (UI/ml)	-0.07	0.57*	1							
BMI (kg/m^2^)	0.01	-0.05	0.44	1						
E2 (pg/ml)	0.14	-0.32	0.26	0.66*	1					
A (nmol/L)	0.22	0.61	0.65*	0.2	0.26	1				
T (nmol/L)	0.01	0.21	0.53*	0.66*	0.72	0.55*	1			
DHEAS (µg/dL)	0.08	0.47	0.83*	0.65*	0.22	0.71*	0.54*	1		
17OHP (ng/ml)	-0.15	0.18	0.52	0.72*	0.77*	0.44	0.74*	0.56*	1	
SHBG (nmol/L)	-0.02	0.06	-0.47	-0.39	0.13	-0.15	0.09	-0.64*	-0.15	1
Months of amenorrhea	-0.02	-0.4	-0.22	-0.11	-0.45	-0.54	-0.23	-0.14	-0.46	-0.22

A negative correlation is denoted by a minus sign, while positive correlations are indicated otherwise

^*^Statistically significant correlations are highlighted in bold (p-value < 0.05)

AMH, anti-Mullerian hormone; FSH, follicle-stimulating hormone; LH, luteinizing hormone; BMI, body mass index; E2, estradiol; A, androstenedione; T,testosterone; DHEAS, dehydroepiandrosterone-sulfate; 17OHP, 17-hydroxyprogesterone; SHBG, sex hormone-binding globulin

**Fig. 3:**
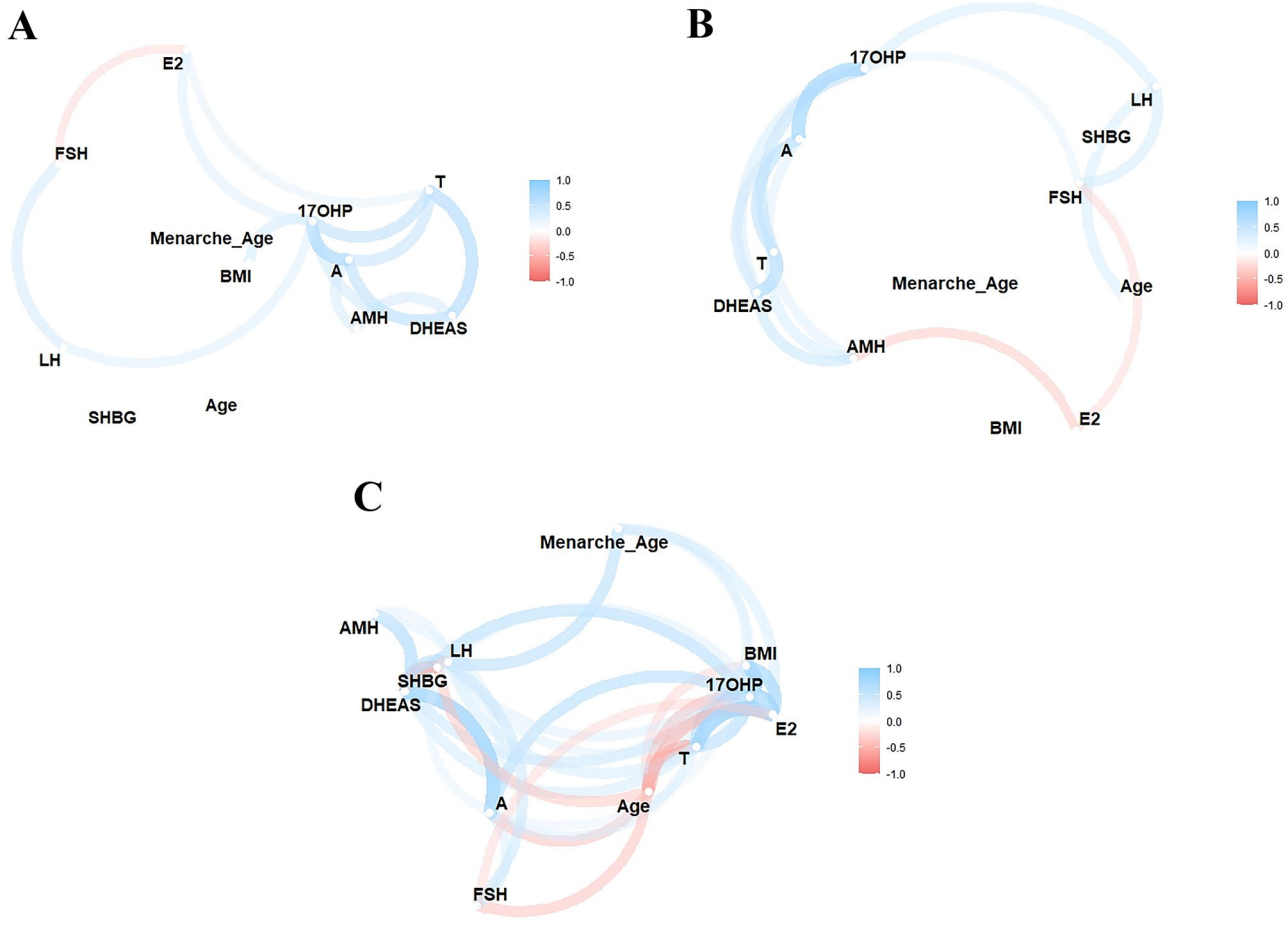
Hormonal correlation networks in controls and FHA subgroups. Network graphs depict pairwise Spearman correlations among hormonal and clinical variables. Blue edges represent positive correlations and red edges represent negative correlations; edge thickness is proportional to correlation strength. Node proximity reflects similarity in correlation patterns. (**A**) Healthy controls. (**B**) Patients with FHA without PCOM. (**C**) Patients with FHA+PCOM. The networks highlight distinct endocrine architectures across phenotypes, with AMH clustering with androgens in FHA without PCOM and showing a more morphology-driven pattern in FHA+PCOM. Abbreviations: FHA, functional hypothalamic amenorrhea; PCOM, polycystic ovarian morphology; AMH, anti-Müllerian hormone; LH, luteinizing hormone; FSH, follicle-stimulating hormone; E2, estradiol; SHBG, sex hormone-binding globulin

Boxplots clearly illustrate the divergent AMH distributions: overall FHA patients did not differ from controls (Fig. 2A), but FHA without PCOM exhibited reduced AMH (Fig. 2B), while FHA+PCOM showed significantly higher levels (Fig. 2C). Network analyses (Fig. 3A–C) were used to visualize the dependency structure among hormonal variables in the three groups and to explore how FHA—particularly its PCOM and non-PCOM phenotypes—modifies endocrine correlations. Each network displays pairwise Spearman correlations as weighted edges, with positive correlations shown in blue and negative correlations in red, and edge thickness proportional to correlation strength. Node proximity reflects the relative similarity and interdependence between hormones within each group. In the overall FHA cohort (Fig. 3A), the network revealed two distinct hormonal clusters. The first cluster comprised gonadotropins (FSH, LH) and estradiol, reflecting their expected physiological interrelations along the hypothalamic–pituitary–ovarian axis. The second cluster grouped AMH with androgens (testosterone, androstenedione, DHEAS), indicating that AMH expression in FHA is more closely aligned with androgen availability than with gonadotropin levels. SHBG was positioned at the periphery of the network with predominantly negative associations, while BMI showed a modest connection with LH. This configuration highlights that, under hypothalamic suppression, AMH becomes functionally linked to androgens, possibly reflecting FSH-independent regulation of early folliculogenesis. In FHA patients without PCOM (Fig. 3B), the network structure became more polarized. The androgen–AMH cluster was preserved, with AMH showing strong positive correlations with testosterone, androstenedione, and DHEAS. Notably, AMH also developed a negative correlation with estradiol, suggesting that reduced estradiol production may be associated with a diminished granulosa-cell environment and consequently impaired AMH expression. Gonadotropins and estradiol formed a compact cluster, illustrating the profound suppression of the gonadotropin–estradiol axis in this phenotype. AMH thus emerged as a ‘bridge’ node connecting the androgenic and hypoestrogenic axes, supporting the interpretation that AMH reduction in FHA without PCOM reflects combined hypogonadotropic and hypoandrogenic states. In the FHA+PCOM group (Fig. 3C), the network architecture shifted substantially toward a PCOS-like endocrine pattern. The androgen cluster strengthened, with dense and strong intercorrelations among testosterone, androstenedione, DHEAS, 17-OHP, and LH. BMI also correlated more tightly with hormonal nodes, particularly LH and estradiol, echoing phenotypes typically observed in PCOS populations. Interestingly, AMH in this group no longer showed strong associations with other hormones and moved toward the periphery of the network, suggesting that its elevation in FHA+PCOM reflects intrinsic follicle excess rather than direct modulation by the hypogonadotropic environment. The resulting structure highlights that FHA+PCOM represents a distinct phenotype in which the underlying ovarian morphology maintains androgen-driven follicular activity despite hypothalamic suppression.

## Discussion

The findings of this study provide new insights into the relationship between AMH levels and FHA, highlighting the heterogeneity of this condition and its clinical implications. The existing literature on AMH in FHA is conflicting. Some studies reported AMH concentrations comparable to those of healthy women [17] or even increased [19] while others described reduced levels [20]. Such inconsistency likely reflects the variability of FHA phenotypes and the influence of ovarian morphology. FHA results from chronic energy deficiency due to insufficient caloric intake, excessive physical activity, psychological stress, or a combination of these factors [15]. Within this heterogeneous population, a subgroup presents with polycystic ovarian morphology (PCOM), a feature usually associated with PCOS. PCOM is characterized by high AMH levels and may represent a distinct phenotype, sometimes referred to as the overlap between FHA and PCOS. This overlap complicates diagnosis and management and highlights the need for careful evaluation of ovarian morphology. It should be noted that although PCOM is often associated with PCOS in adults, the presence of PCOM alone is not considered a diagnostic criterion in adolescents according to the most recent international guideline [25]. In our cohort, 28% of FHA patients exhibited PCOM, consistent with previous reports [22]. When the FHA group was analyzed as a whole, AMH levels were similar to those of controls. However, stratification revealed that FHA without PCOM was associated with significantly lower AMH, while FHA with PCOM showed increased values, mimicking the pattern of PCOS. This observation supports the hypothesis that ovarian morphology may play a critical role in AMH secretion under hypothalamic suppression. Importantly, the duration of amenorrhea did not differ between FHA phenotypes and was not associated with AMH concentrations. This finding supports the interpretation that reduced AMH levels in FHA without PCOM reflect a functional and potentially reversible suppression of folliculogenesis rather than a cumulative effect of prolonged hypothalamic dysfunction. Our analysis revealed that patients with PCOM had a higher BMI compared to patients with FHA alone. Interestingly, unlike the latter group, the BMI of patients with PCOM did not show a significant difference from the control population. Additionally, the reduction in androgens was less pronounced in patients with PCOM, with testosterone being the only hormone affected. This suggests that some women with FHA and PCOM may have had an underlying PCOS phenotype, subsequently masked by hypothalamic dysfunction triggered by energy deficit. In this subgroup, we observed a positive correlation between BMI and estradiol, a pattern consistent with what has been described in PCOS populations. The association between higher BMI and elevated estrogen levels is well documented [26] and is largely explained by the aromatization of androgens into estrogens within adipose tissue, particularly visceral fat [27]. As a result, women with greater adiposity tend to exhibit increased estrogen production. PCOS, which is strongly linked to obesity, is frequently characterized by hyperestrogenism and chronic anovulation [28]. By contrast, in the overall FHA group, estradiol did not correlate with BMI; instead, BMI correlated positively with LH. This finding reflects the complex regulation of the HPG axis in FHA and PCOS. In obesity, adipokines and insulin resistance disrupt LH secretion [29] while in energy deficit and low adiposity, GnRH pulsatility and LH release are suppressed [30]. Adipocytokine changes such as increased adiponectin, ghrelin, and PYY, along with reduced leptin, contribute to this suppression [31]. In our cohort, FHA patients had lower BMI and reduced gonadotropins, estrogens, and androgens compared with controls, consistent with hypothalamic dysfunction. Interestingly, we found a positive correlation between AMH and androgens, rather than with gonadotropins. While some studies reported associations between reduced AMH and gonadotropin deficiency [30], our data highlight a potential role of androgens in AMH regulation. Evidence from PCOS studies shows that androgens enhance FSH receptor expression in granulosa cells, amplifying AMH production during early folliculogenesis [32]. In FHA, reduced LH secretion decreases theca cell androgen synthesis [20], potentially attenuating this synergy. Androgen deficiency may therefore weaken the stimulatory effect of FSH on granulosa cells [31], further reducing AMH production in addition to the effects of relative FSH deficiency. We also observed an inverse correlation between AMH and estradiol. Although seemingly paradoxical, this finding is consistent with data from the ovulatory cycle, where estradiol suppresses AMH expression in granulosa cells via estrogen receptor (ER) β [32]. By contrast, ERα-mediated signaling in the corpus luteum may stimulate AMH [32]. The network analyses highlight that AMH is embedded within different endocrine architectures depending on FHA phenotype. In FHA without PCOM, AMH clusters with androgens and away from suppressed gonadotropins, supporting the idea that reduced AMH reflects functional suppression rather than impaired ovarian reserve. In FHA+PCOM, the network becomes more PCOS-like, with AMH positioned as a morphology-driven marker, relatively independent of gonadotropin suppression. These patterns indicate that AMH regulation in FHA is phenotype-specific These patterns suggest that FHA should not be regarded as a uniform disorder but may represent a spectrum of phenotypes with distinct endocrine signatures. Identifying such variability may help refine clinical classification and guide tailored management.

### Limitations of the study

This study has some limitations. The sample size was relatively small, partly due to the strict age-matching with controls, which reduced the pool of eligible participants. A further limitation is the absence of formally reported AFC values. Since ultrasounds were performed as part of routine clinical practice rather than within a standardized research protocol, AFC was not numerically documented, although follicle number was always assessed and used for PCOM classification. This approach may have restricted the detailed morphological characterization of the ovarian reserve. Although ultrasound assessments were performed by only three experienced operators who were trained within the same institution and adhered to identical scanning protocols, formal intra-observer and inter-observer reproducibility was not evaluated. This represents a methodological limitation that may introduce a degree of variability in PCOM assessment, despite the standardized approach. Another limitation of this study is the absence of systematic quantitative data on lifestyle determinants such as exercise load, caloric intake, and psychological stress. Although all participants underwent initial clinical, nutritional, and psychological evaluations, these variables were not collected using standardized instruments. Given their central etiological role in FHA, the lack of detailed lifestyle metrics may limit the ability to correlate the degree of hypothalamic suppression with the observed endocrine profile. These limitations should be considered when interpreting our findings.

## Conclusions

In summary, our findings suggest that reduced AMH levels in FHA may primarily reflect hypothalamic suppression of gonadotropins rather than an irreversible depletion of the ovarian reserve. This observation has important clinical implications: in these patients, AMH may underestimate reproductive potential, leading to misleading fertility counseling. We propose that AMH in FHA should be interpreted with caution, as recovery of hypothalamic–pituitary activity and resumption of menstrual cycles may restore gonadotropin secretion and, consequently, AMH production. Future longitudinal studies are warranted to confirm whether AMH levels increase with cycle recovery and to determine their predictive value for fertility outcomes in this population.

## Data Availability

Source data for this study are available at https://redcap-irccs.policlinicogemelli.it. The datasets generated and/or analyzed during the current study are available from the corresponding author on reasonable request.
